# Comparison of Pattern Detection Methods in Microarray Time Series of the Segmentation Clock

**DOI:** 10.1371/journal.pone.0002856

**Published:** 2008-08-06

**Authors:** Mary-Lee Dequéant, Sebastian Ahnert, Herbert Edelsbrunner, Thomas M. A. Fink, Earl F. Glynn, Gaye Hattem, Andrzej Kudlicki, Yuriy Mileyko, Jason Morton, Arcady R. Mushegian, Lior Pachter, Maga Rowicka, Anne Shiu, Bernd Sturmfels, Olivier Pourquié

**Affiliations:** 1 Stowers Institute for Medical Research, Kansas City, Missouri, United States of America; 2 Theory of Condensed Matter, Cavendish Laboratory, Cambridge, United Kingdom; 3 Department of Computer Science, Duke University, Durham, North Carolina, United States of America; 4 Department of Mathematics, Duke University, Durham, North Carolina, United States of America; 5 Geomagic, Research Triangle Park, North Carolina, United States of America; 6 INSERM U900, Paris, France; 7 CNRS UMR 144 Curie Institute, Paris, France; 8 Ecole des Mines de Paris, Paris Tech, Fontainebleau, France; 9 Department of Biochemistry, University of Texas Southwestern Medical Center, Dallas, Texas, United States of America; 10 Department of Mathematics, University of California, Berkeley, California, United States of America; 11 Howard Hughes Medical Institute, Kansas City, Missouri, United States of America; University of Glasgow, United Kingdom

## Abstract

While genome-wide gene expression data are generated at an increasing rate, the repertoire of approaches for pattern discovery in these data is still limited. Identifying subtle patterns of interest in large amounts of data (tens of thousands of profiles) associated with a certain level of noise remains a challenge. A microarray time series was recently generated to study the transcriptional program of the mouse segmentation clock, a biological oscillator associated with the periodic formation of the segments of the body axis. A method related to Fourier analysis, the Lomb-Scargle periodogram, was used to detect periodic profiles in the dataset, leading to the identification of a novel set of cyclic genes associated with the segmentation clock. Here, we applied to the same microarray time series dataset four distinct mathematical methods to identify significant patterns in gene expression profiles. These methods are called: Phase consistency, Address reduction, Cyclohedron test and Stable persistence, and are based on different conceptual frameworks that are either hypothesis- or data-driven. Some of the methods, unlike Fourier transforms, are not dependent on the assumption of periodicity of the pattern of interest. Remarkably, these methods identified blindly the expression profiles of known cyclic genes as the most significant patterns in the dataset. Many candidate genes predicted by more than one approach appeared to be true positive cyclic genes and will be of particular interest for future research. In addition, these methods predicted novel candidate cyclic genes that were consistent with previous biological knowledge and experimental validation in mouse embryos. Our results demonstrate the utility of these novel pattern detection strategies, notably for detection of periodic profiles, and suggest that combining several distinct mathematical approaches to analyze microarray datasets is a valuable strategy for identifying genes that exhibit novel, interesting transcriptional patterns.

## Introduction

The dynamics of gene expression in a biological system exposed to varying experimental conditions, such as dose response to a drug or a time course, can be analyzed now at the whole genome level by generating series of microarrays or using massively parallel sequencing technologies. Each gene in the genome becomes associated with a set of expression values, called gene expression profile. The main challenge for the biologist is to identify, among the tens of thousands of gene expression profiles, trends or patterns revealing biological properties of the system that may lead to the formation of novel hypotheses. Some such patterns are easy to detect, e.g., when a gene is silent under most conditions but is actively transcribed under a subset of conditions. However, other patterns may be subtle and of unknown shape, as well as relatively noisy, so there is a continuous need for better methods of pattern detection in gene expression data.

Microarray time series have been extensively generated to study periodic biological processes, such as the cell cycle [1], circadian regulation [2], [3], the life cycle of malaria parasite in human blood [4] and vertebrae segmentation [5]. In most of these cases, the periodic behavior observed at the macroscopic scale is associated with periodic changes in the level of multiple mRNAs. Several approaches have been used to identify genes whose periodic expression underlies the cellular- or tissue-level periodic behavior of the system. A common feature of these approaches is their strict assumptions about the shape of periodic profiles. For example, popular Fourier-based methods detect periodicity by decomposing gene expression profiles into a series of sine curves. However, these methods are less sensitive to many types of periodic profiles that are poorly approximated by sine curves (because of the noise in the experimental measurements or because periodic profiles might have a different shape, such as asymmetric profiles with short peak and long trough), introducing biases to the results. Moreover, little attention has been given to the possible presence of aperiodic, yet non-random, patterns of gene expression in the transcription program of periodic biological processes.

The segmentation of the vertebrate axis into periodic structures, such as vertebrae, occurs during embryogenesis when the vertebral precursors, the somites, are formed rhythmically from the presomitic mesoderm (PSM). This process is associated with a molecular oscillator, the segmentation clock, which drives periodic gene expression in the PSM with a period corresponding to that of somite formation [6], [7]. During one somite formation (one clock cycle), cyclic genes, such as *Lunatic fringe* (*Lfng*), are expressed as a wave initiated in the posterior PSM that progressively migrates along the PSM and narrows as it moves anteriorly [8]–[10]. A microarray time series of PSM samples encompassing one period of the segmentation clock has been generated in the mouse and analyzed using the Lomb-Scargle (L) periodogram, a method related to Fourier analysis in that it attempts to fit the observed data to a sine curve [5], [11] (Microarray data are available at ArrayExpress at www.ebi.ec.uk/arrayexpress/ under accession number E-TABM-163). This analysis identified a large number of novel cyclic genes that fall into two biologically coherent clusters oscillating in opposite phase, one of which is associated with the Wnt and the other with the Notch and FGF signaling pathways.

This paper is the result of a collaborative effort which occurred in the context of the Defense Advanced Research Projects Agency (DARPA) FUNBIO program that brought together mathematicians, physicists and biologists to evaluate novel mathematical approaches for biological data analysis. In this paper, we applied four different mathematical approaches to the same mouse segmentation dataset and compared the results to the original study. The four methods are: Phase consistency (P), Address reduction (A), Cyclohedron test (C), and Stable persistence (S). These methods can be divided into two groups. In the first group, the P and S methods [12] are hypothesis-driven and search for periodic profiles but in a very different way compared to the L method. The P method optimizes the ratio of the total variation to the sum of the piecewise variations, with the pieces set by the behavior of the known cyclic gene *Lfng*, and the S method is based on a numerical assessment that is provably stable (see Materials and Methods - “Pattern Detection Methods”). In the second group, the A [13], [14] and C [15], [16] methods are data-driven and attempt to identify significant patterns without assuming the periodic nature of the patterns of interest. Both methods associate significance inversely with the likelihood of certain groups of patterns but differ from each other in how they partition the set of all possible patterns into groups.

All methods identified previously known cyclic genes among their top ranked candidates and each method identified a number of novel candidate cyclic genes of the Wnt pathway. We show that one such gene, coding for the Wnt-target and Wnt-modulator *cysteine rich protein 61* (*Cyr61*) identified by three of the methods, represents a novel bona fide cyclic gene of the mouse segmentation clock.

## Results and Discussion

In this study, we used a microarray dataset generated in earlier work [5] to identify cyclic genes associated with the mouse segmentation clock. A microarray time series was generated by collecting the PSM tissue from 17 embryos (17 time points) along the clock cycle and analyzed using L analysis [5] that focuses on the genes whose expression patterns display the best fit to a sine curve [11]. This led to identification of 27 strongly periodic probe sets (corresponding to 25 genes), including seven cyclic genes whose cyclic expression pattern had been discovered earlier by *in situ* hybridization [8]–[10], [17]–[22] and 20 more probe sets that subsequently were experimentally validated by *in situ* hybridization after L analysis [5] (Supplementary Information, Table S1).

**Figure 1:**
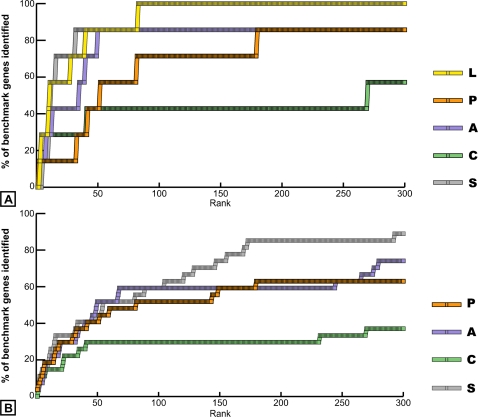
Identification of benchmark cyclic genes in the top 300 probe set lists of the five methods. (A) Benchmark genes are composed of cyclic genes identified independently from the Lomb-Scargle (L) analysis (seven probe sets). (B) Benchmark genes also include cyclic genes identified by the L analysis and experimentally validated (27 probe sets). L, Lomb-Scargle analysis; P, Phase consistency; A, Address reduction; C, Cyclohedron test; S, Stable persistence.

In this study, we used the four methods (P, A, C and S) to rank the 7,549 probe sets of the same dataset in order of the significance of their expression profile (as defined by each method (see Materials and Methods - “Pattern Detection Methods”). We selected the top 300 ranked probe sets from each list (Supplementary Information, Tables S2, S3, S4, S5 and S6). First, we compared the rank of the seven known cyclic genes that were independently identified from non-microarray experimental methods. The L, P, A and S methods each identified at least five out of the seven benchmark genes in the top ranked 100 probe sets; whereas, C identified three of the seven known genes (Figure 1A). To measure the performance to a higher resolution, we repeated this analysis using a larger collection of 27 probe sets experimentally validated [5]. As indicated in Figure 1B, method S performs best by identifying 90% of the benchmark probesets, followed by methods A and P (approximately 75% and 63%, respectively) and C (approximately 37%). Among the top 10 probe sets of each list, the methods perform similarly by ranking among them from four to five benchmark probesets. Thus, all methods, whether or not designed to detect specifically periodic patterns, identified cyclic genes from among their top ranked candidates. This suggests that periodic patterns of gene expression are predominant among all non-random patterns in this dataset, which is consistent with the experimental design of the time series generation. Indeed, due to technical issues, the right PSM samples of the time series were dissected from mouse embryos belonging to five consecutive somite cycles, and they were ordered based on their phase of *Lfng* expression pattern (revealed by *in situ* hybridization on the left PSM of each dissected mouse embryo) to reconstitute a unique oscillation cycle [5]. One of the consequences of this strategy is that the collapsed dataset generated by this procedure preserves periodic patterns associated with the segmentation clock, while it may affect most other patterns (such as a linear increase with developmental time).

**Figure 2:**
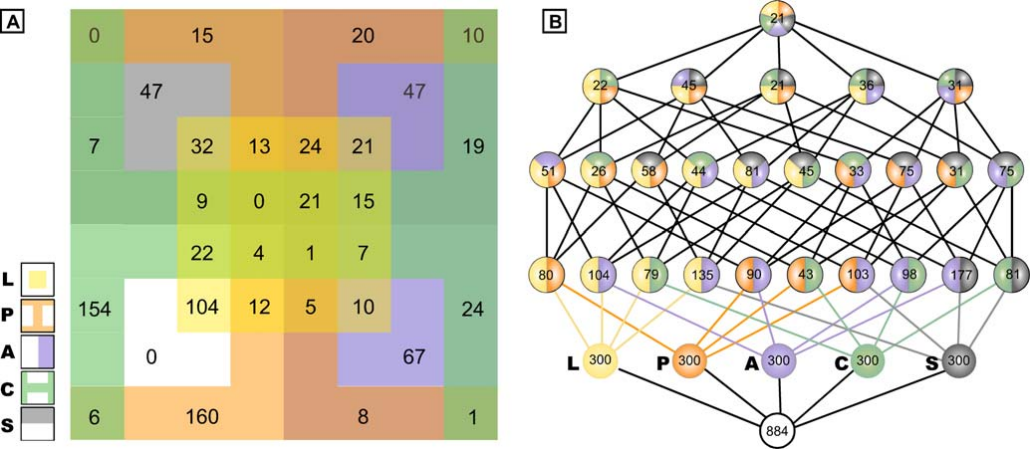
Comparison of the intersection of the top 300 ranked probe sets from the five methods. (A) Venn diagram. (B) Haase diagram shows the pairwise intersection of two lists, the triple intersection of three lists, and so on. The total number of distinct probe sets in all of the five top 300 lists (the union) is 884; the total number in each of the five sets (the intersection) is 21. L, Lomb-Scargle analysis; P, Phase consistency; A, Address reduction; C, Cyclohedron test; S, Stable persistence.

We next compared the intersection of the top 300 ranked probe sets from the four methods and method L. This is represented in Figure 2A as a five-set Venn diagram in which each color corresponds to a different method and in Figure 2B as a Haase diagram in the form of the lattice of the subsets of a five-element set. The total number of distinct probe sets in all of the five sets (the union) is 884; the total number in each of the five sets (the intersection) is 21. The overlap contains eight true positive cyclic genes (Supplementary Information, Table S7). Many candidate genes were identified by only one, two, three or four methods. The L, P and C methods identified larger numbers of unique genes (104, 160 and 154, respectively) compared to method A (67) and method S (47) (Figure 2). Although it is not possible to know whether all the uniquely predicted genes are associated with the segmentation clock, many of them are biologically plausible since they are associated with the Wnt pathway.

**Figure 3:**
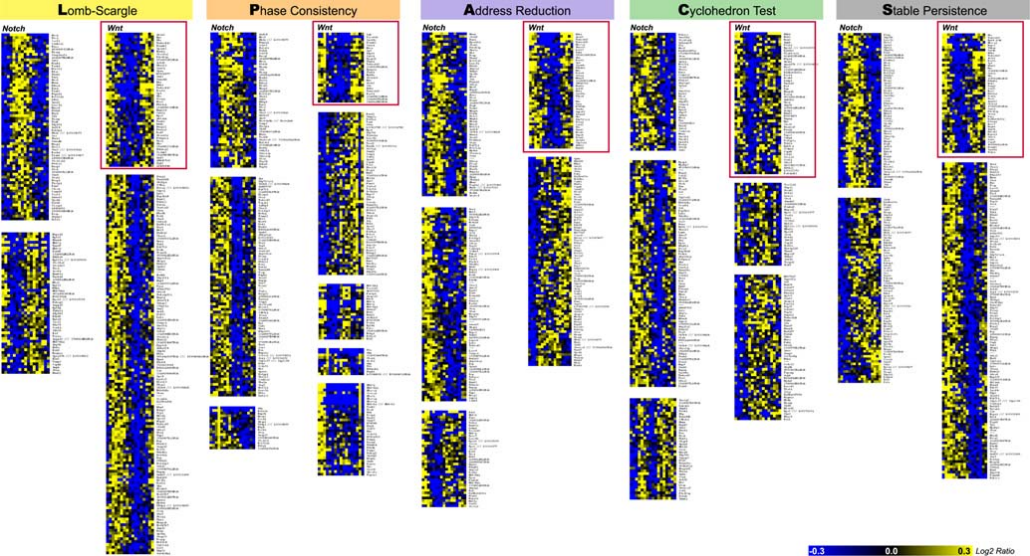
Clustering analysis of the top 300 ranked probe sets from the five methods. K-means clustering was applied as described in the Materials and Methods section. The “Notch” and “Wnt” clusters contain validated cyclic genes regulated by the Notch and Wnt pathways, respectively. Blue, decrease in gene expression; yellow, increase in gene expression. Wnt clusters are boxed in red.

We then studied the possible biological links of the top 300 genes predicted by each method to the segmentation clock process. Most of the validated cyclic genes (22 probesets or 20 genes) identified in the top 38 probesets list of the original L analysis are associated with the Notch-, FGF- and Wnt-signaling pathways and organized in two clusters [5]. In the first cluster, almost one half of the genes are associated with the Notch- and FGF-signaling pathways. The second cluster shows an even more striking biological coherence with more than 90% of the genes linked to Wnt signaling. To test the biological relevance of the predictions of each method, we investigated their ability to identify novel cyclic candidates associated with Wnt signaling. We first independently clustered the top 300 gene expression profiles identified by the P, A, C, S and L methods (Figure 3; Supplementary Information, Table S8, S9, S10, S11 and S12 and Materials and Methods). While the L and S methods identified periodic patterns oscillating in different phases, the P, A and C methods also identified a larger variety of patterns (illustrated by higher numbers of clusters). Dimensionality analysis, inferred from principal component analysis (Materials and Methods; Figure S1) of the corresponding top 300 probeset lists, is in agreement with these observations. The highest numbers of degrees of freedom (between four and six) found for C and A stem from the uninformed priors used in these methods, allowing for discovery of unrestricted profile shapes. In contrast, the other methods are characterized by smaller numbers of degrees of freedom: L had only two significant degrees of freedom, corresponding to sine and cosine of the main harmonic oscillation; S had two strong sin-cos components, plus two additional, presumably related to shape parameters; and finally, P showed three degrees of freedom (corresponding to the difference between phases 1 and 3, the difference between phases 2 and 3, and the variation within phase 3).

**Figure 4:**
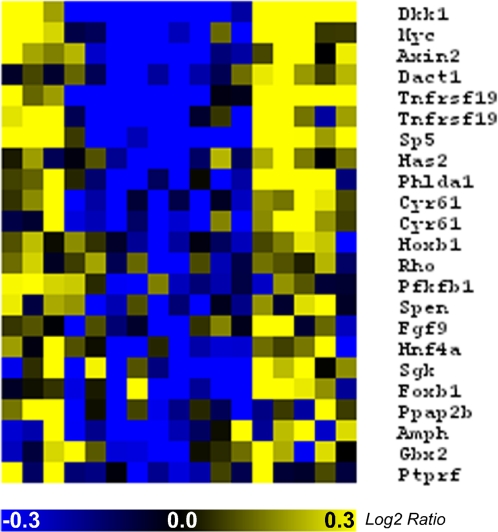
Heatmap of the members of the Wnt clusters identified by the five methods. Blue, decrease in gene expression; yellow, increase in gene expression.

In each of the five analyses, we identified a cluster of periodic profiles containing the known cyclic genes *Axin2*, *dickkopf homolog 1* (*Dkk1*), *myelocytomatosis oncogene* (*c-Myc*) and *dapper homolog* (*Dact1*) of the Wnt pathway (Figure 3 [red boxes]; Figure 4; Table 1). Given the very tight biological coherence of the Wnt cluster identified by the L method and according to the principle of “guilt by association,” it is likely that other gene members of the cluster also belong to the Wnt pathway. We find that the S method identifies the eight known members of the Wnt cluster previously identified by the L method and validated as described [5], while the P and A methods identify six out of eight, and the C method identifies five out of eight of the known Wnt cyclic genes. We further analyzed the novel candidate cyclic genes contained in each of the Wnt clusters through a literature search to investigate their potential link to Wnt signaling (Table 1). The PubMed database was searched for each of the 142 genes in the Wnt clusters to identify articles indicating a link between these genes and the Wnt pathway. The results indicated some articles containing the two search terms: the “gene name” and “Wnt.” Manual curation of these results was necessary to verify the biological connection between each gene and the Wnt pathway. The search of the PubMed database was automated using the MedlineR library [23] for the R statistical language. The number of matches for each pair of search terms was returned, as well as a link to the abstracts for each match. Thirteen genes were identified as novel Wnt cyclic gene candidates (Table 1).

**Table 1:** Composition of the Wnt Clusters of the Five Methods.

Predicted Wnt-Cyclic Genes		Gene Symbol	L Rank	P Rank	A Rank	C Rank	S Rank
Wnt-benchmark cyclic genes		*Dkk1*	1	1	1	1	1
		*Myc*	1	1	1	1	1
		*Axin2*	1	1	1	1	1
		*Dact1*	1	1	1	1	1
		*Tnfrsf19*	2	2	2		2
		*Sp5*	1	1	1		1
		*Has2*	1			1	1
		*Phlda1*	1				1
Wnt-candidate cyclic genes	Candidates identified by several methods	*Cyr61*	2	2			1
		*Hoxb1*	1		1		
		*Rho*	1		1		
		*Pfkfb1*			1		1
	Candidates identified by only one method	*Spen*	1				
		*Fgf9*	1				
		*Hnf4a*		1			
		*Sgk*		1			
		*Foxb1*		1			
		*Ppap2b*		1			
		*Amph*				1	
		*Gbx2*				1	
		*Ptprf*				1	

Numbers indicate the count of probe sets detected per gene.

L = Lomb-Scargle analysis; P = Phase consistency; A = Address reduction; C = Cyclohedron test; S = Stable persistence.

This allowed us to identify six novel candidate genes in the L-Wnt cluster showing a link to the Wnt pathway. These include the genes *Cyr61* [24], *homeo box B1* (*Hoxb1*) [25], *rhodopsin* (*Rho*) [26], *SPEN homolog*, *transcriptional regulator* (*Drosophila*), (*Spen*) [27] and *fibroblast growth factor 9* (*Fgf9*) [28] which were all previously identified as Wnt targets. Interestingly, the P method performs similarly to the L method by predicting six new candidates (although only two are common with those predicted by L), while A, S and C methods predict two, two and three additional members, respectively. Most of the Wnt cyclic gene candidates predicted by the L method are also predicted by at least one of the other methods. In contrast, the P and C methods identify the largest numbers of putative Wnt pathway candidates that are not predicted by any other method.

**Figure 5:**
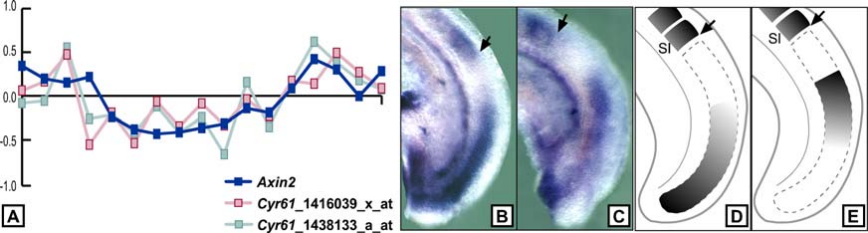
Identification of *Cyr61* as a novel Wnt-cyclic gene. (A) Gene expression profiles (in log_2_ ratio of *Cyr61*) represented by two probe sets and the benchmark Wnt-cyclic gene *Axin2*. (B–E) Experimental validation by *in situ* hybridization. (B, C) Lateral views of the tails of 9.0 dpc mouse embryos hybridized with the *Cyr61* probe. (D, E) are schematic representations of (B) and (C), respectively, and illustrate the dynamic expression of the gene in the presomitic mesoderm. Arrows in all four panels indicate the posterior boundary of the last formed somite (SI).

One of the candidates, *Cyr61* (Figure 5A–E), a well known Wnt target [29] which codes for a modulator of the Wnt-signaling pathway [24] that had not been identified in the original analysis [5], was identified by three of the methods. We experimentally investigated the expression pattern of this gene by *in situ* hybridization in the PSM of mouse embryos (Figure 5B–E) and indeed, observed a dynamic pattern of expression in the posterior PSM that was reminiscent of the typical expression of a cyclic gene; hence, validating it as a novel cyclic gene and extending the list of known cyclic genes associated with the Wnt pathway. More generally, the other Wnt cyclic gene candidates identified by the different methods are attractive, potential new cyclic genes involved in the mouse segmentation clock and would deserve to be further experimentally validated. Interestingly, the *gastrulation brain homeobox 2* (*Gbx2*) gene predicted by method C and which is a target of Wnt signaling, was reported to be expressed in the PSM [28], [29].

An interesting feature common to the A, C and S methods is that they work on the ranked data as opposed to the raw amplitudes of the signal. In other words, the signal intensities that describe over time the expression of a gene are sorted by magnitude, and each signal intensity is then replaced by the integer rank within this sorted order. Thus, each gene expression profile is represented by a permutation that is invariant over the transformations of monotonically increasing functions (such as log or taking the square) and is much more mathematically tractable. These methods offer a particular advantage for the analysis of this segmentation clock time series in which only the ordering of the time points could be estimated, but not the exact time interval in between. These methods, based on the rank permutation, do not require such precise timing information. These methods perform similarly to the L and P methods, which use the raw signal intensities. This suggests that moving to ranked data despite losing some information (like fold change), might be advantageous in certain cases, especially when these methods are not based on *a priori* biological knowledge, making them promising exploratory tools to discover novel, interesting transcriptional patterns in large-scale expression analysis.

## Materials and Methods

### Description of the starting microarray dataset

Microarray data, available at ArrayExpress at www.ebi.ec.uk/arrayexpress/ under accession number E-TABM-163, were normalized as described [5] and filtered based on detection call (by removing the probe sets called “absent” throughout the experiment), signal intensity (by removing genes with low-maximum expression level <50) and amplitude (by eliminating peak-to-trough variation <1.65). After these filters, the dataset consisted of 7,549 probe sets.

### Pattern Detection Methods

Here we describe the four methods used in the analysis of the mouse segmentation clock: Phase consistency (P), Address reduction (A), Cyclohedron test (C) and Stable persistence (S). For further details, we refer the reader to papers describing each individual method. A description of the Lomb-Scargle method (L), previously used to study the mouse segmentation clock in [5] can be found in [11]. In contrast to experiments exhibiting two or more periods, which are well suited to Fourier analysis and related methods (see, e.g.,[30]), the mouse embryo data in this paper represents only a single period. The analysis of this system is therefore particularly challenging and requires novel approaches.

The methods A, C and S begin by converting the raw data into rank order, which we explain here. Each gene is characterized by its expression profile, which is a function *f* whose values are given at *N*  =  17 distinct time points. In the raw data, these values *f_i_*, for 1≤*i*≤*N*, are real numbers quantifying the amount of expression as measured by the microarray. We sort the values and replace each *f_i_* by its rank within this sorted order. For example, the values (0.41, 0.63, 0.11, 0.23, 0.59) would be replaced by (3, 5, 1, 2, 4). The function *f* is thus replaced by a permutation which we denote *π*(*f*) and the *i*th element of *π* is *π_i_*. Ramifications of this step are provided in the Discussion.

All four methods associate with each gene expression profile a number *μ*(*f*). This number is used to rank the probe sets in order of significance (as defined by each method).

#### Phase consistency (P)

This method is unique in that it looks directly at the raw data (*f* rather than *π*(*f*)) and uses information that is specific to the mouse embryo experiment. Specifically, it was observed that the expression of the gene *Lfng* suggests a decomposition into three *phases* comprising the first four, the next five and the last eight measurements in each time series. To make the phase lengths more equal in size, we further divided the last phase into two subphases, each comprising four measurements. Measurements were assigned to the respective subphases according to their projection onto the main principal component of the data in the eight-dimensional space. The periodicity of a function *f* is assessed by comparing the overall standard deviation with the sum of the normalized standard deviations of the four phases.

*Mathematical description*— First we normalize the function values to zero average and unit variance: *x_i_* = (*f_i_*−avg)/var^1/2^, where 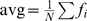 and 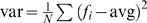. By construction, the standard deviation of the normalized values is 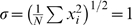. The estimated population standard deviations of the four phases are denoted by *σ_i_*. The measure used by P is then
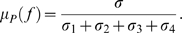
Ordering by decreasing *μ_P_* prioritizes profiles which exhibit a high global variation and low local variation.

#### Address reduction (A)

This method is based on the idea of algorithmic information content, also known as Kolmogorov complexity. In its original form, the Kolmogorov complexity is the length, in bits, of the shortest description of a data string given some universal computer. Address reduction bounds the Kolmogorov complexity of a gene expression profile and uses this to determine how much a curve can be compressed, measured in bits. This method works with the ranked data *π*(*f*), and the bound is calculated by dividing the address of the rank permutation into two parts: a coarse address (a block) and a fine address (the permutation within the block).

**Figure 6:**
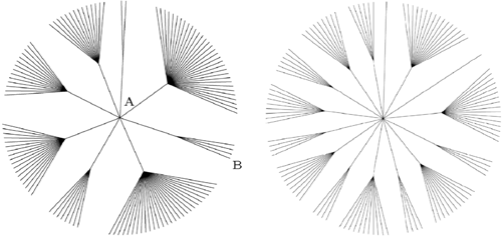
Address reduction. The tree representations of the block structures for the blocking functions *γ*_Δ1_ (left) and *γ*_+−_ (right) (*γ*_+−_ is the number of permutations with a given sequence of rises and falls [14]). Locating a given curve using the two-part address is equivalent to starting at the centre of the tree (A) and finding a particular exit at the edge (e.g., B). Address reduction *μ_A_* gives the reduction in information, measured in bits, to get from A to some B, compared to the information needed to locate B explicitly. In the case above, the endpoint B, being in a block of four, could correspond to the permutation (4, 5, 3, 2, 1) (this and three other permutations have *γ*_Δ1_  =  5). To find it, someone starting at A would require log_2_ 8+log_2_ 4 = 5 bits of information (8 paths to choose from, then 4 paths to choose from), which is *μ_A_*  =  1.91 bits less than that required to transmit (4, 5, 3, 2, 1) explicitly, namely, log_2_ 5!.

*Mathematical description*—We first partition the space of permutations into blocks using some *blocking function γ_A_* that maps each permutation to a real number; permutations with the same number are in the same block. Second, we base the measure of an expression profile *f* on the size of the block that contains it, and the total number of possible blocks. Then the number of bits *μ_A_*(*f*) that *f* can be compressed is
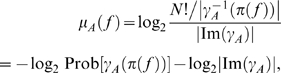
where 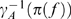 is the set of permutations mapping to the same block as *π*(*f*) and Im(*γ_A_*), the image of *γ_A_*, is the set of possible values that the blocking function can take on. The vertical bars denote the number of elements in the set shown between them. Subtracting the logarithm of the image size allows comparison between different blocking functions and curves with different numbers of data points, which we do not consider here. In the application to the mouse embryo data, we use what is sometimes called the *bounded variation*, 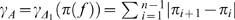, where *π_i_* is the rank of *f_i_* in the sorted order (see Figure 6 left for an example). Other blocking functions can be used: see Figure 1 right and [31], as well as the discussions of the methods C and S. Further details about address reduction can be found in [14], [31].

#### Cyclohedron test (C)

This method is a non-parametric test that determines the significance of each expression profile based on its topography. In addition, *p-*values are computed for groups of highest ranked genes. Similar to A, the score in C is derived from the ranked data *π*(*f*). It involves a partition of the set of patterns, and ascribes higher biological significance to patterns in small blocks of the partition. However, the details of C and A are quite different.

**Figure 7:**
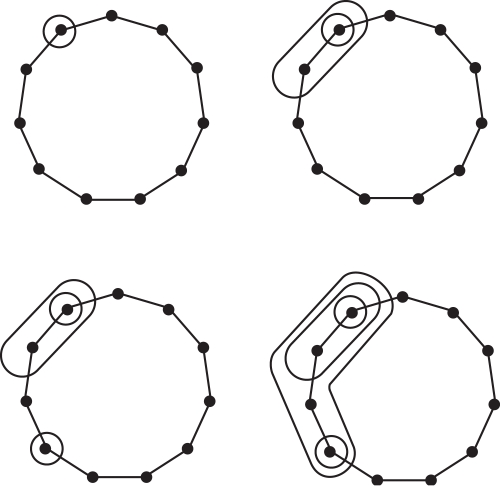
The Cyclohedron test constructs a topographic map on the *N*-cycle by subsequently encircling vertices. Displayed at the top are the formations of the first two circles *σ_i_*, and at the bottom are the third and fourth, for an example with *N*  =  11.

*Mathematical description*—This method is a non-parametric test that is based on the significance of a *topographical map* constructed from a permutation. The topographical map is obtained by encircling the vertices of a cycle in decreasing order of their corresponding raw data vector coordinates. Denoting the first circle by the set *σ*_1_, the second by *σ*_2_, and so on, the circles are constructed according to the following provision: in order to encircle the vertex *δ_i_*, if it is adjacent to some vertex *j* which has already been encircled by some *σ_k_*, then *σ_i_* must contain the *σ_k_* circle. Figure 7 depicts the beginning of the creation of such a topographical map.

The score *μ_C_*(*f*) assigned to the raw data vector *f* is the number of permutations that have the same topographical map as the permutation *π*(*f*); this score is called the *permutation count*. Data vectors with small permutation counts are deemed significant, because it is unlikely that a random permutation will have a topographical map shared by few permutations. A full description of the cyclohedron test, including a method for computing *p-*values, appears in [15]. Connections to algebraic combinatorics are discussed in [16].

#### Stable persistence (S)

In contrast to A and C, this method assesses the biological significance of an expression pattern directly from the corresponding permutation *π*(*f*), without calculating block sizes of the implied partition. The main focus is to use a measure that is stable under small perturbations of the gene expression profile.

*Mathematical description*—Each expression pattern corresponds to one period of somitogenesis. We represent this period by a circle and denote time points where expression levels were measured by *x_i_*, 1≤*i*≤*N*. Since these times are unknown, we choose the *N* points *x_i_* at regular intervals during the period, that is, 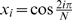, and define *g*(*x_i_*) = (*π_i_*−1)/(*N*−1), where *π_i_* is the rank of *f_i_* in the sorted ordering, as before. Values of *g*(*x*) at other points of the circle are obtained by linear interpolation. Notice that the normalization constant 1/(*N*−1) guarantees that 0≤*g*(*x*)≤1.

**Figure 8:**
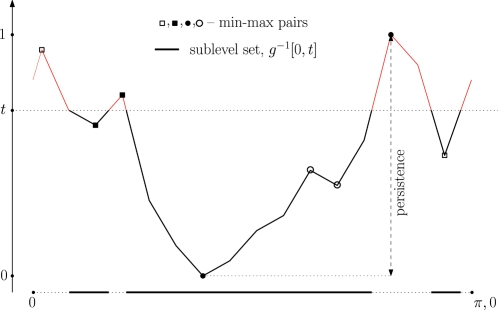
Function *g*(*x*) for the expression pattern of Axin2.

Given a threshold *t*, the *sublevel set g* ^−1^[0, *t*] consists of all points *x* of the circle with *g*(*x*)≤*t*. In other words, it contains all the time intervals during which the (modified) expression level is below *t*. As we increase the threshold from 0 to 1, the sublevel set grows until it eventually covers the entire circle. A *birth event* corresponds to *t* passing the function value of a minimum, at which time a new interval is added to the sublevel set. A *death event* corresponds to *t* passing the value of a maximum, at which time the sublevel set merges two intervals into one or, at the last and global maximum, it closes up to form the complete circle. Using this process we form a canonical pairing in which each minimum is matched with the maximum that merges its interval with another interval started by an earlier minimum; see [12] and Figure 8.

After matching the global minimum with the global maximum we exhausted all minima and maxima and completed the construction of the canonical pairing. The *persistence* of a min-max pair (*x_i_*, *x_j_*) is the difference between the function values pers(*i*, *j*)  =  *g*(*x_j_*)−*g*(*x_i_*), which is necessarily positive. Pairs with small persistence correspond to small local variations of the expression profile, while pairs with large persistence correspond to significant oscillations. We note that the sum of persistences is equal to half the bounded variation used in A, except that there the domain is taken as the interval rather than the circle. For each non-negative integer *p*, we define the *order-p measure* as the sum of the *p*-th powers of the persistences over all min-max pairs. (Incidentally, the blocking functions *γ_A_* and *γ_C_* are related to the order-1 measure.) As proved in [32], this measure is stable for *p*≥2 and unstable for *p* equal to 0 or 1. Method S uses the order-2 measure, since two is the smallest integer power that implies stability. Thus,
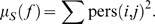
Further details about stable persistence can be found in [12], [32].

### Cluster analysis

The 300 top ranked expression profiles of each method were clustered using K-means based on the Pearson correlation distance in MultiExperiment Viewer (MEV) software. The optimal number of clusters was determined using the Figure of Merit (FOM) function [33] in the MEV package that provides a measure of the fit of the expression patterns for the clusters produced by K-means.

### Dimensionality analysis

Effective dimensionality, or number of degrees of freedom of the five “top 300” datasets (Figure S1) can be inferred from Principal Component Analysis (see e.g., [34]). The transcription profiles have been normalized to zero average and unit variance. Then, principal components have been computed for each of the sets of 300 points (top 300 from each method). Intrinsic dimensions in each set were considered significant until differences in log residual variance drop and converge towards the common noise level.

### Experimental validation

The candidate cyclic gene *Cyr61* was experimentally validated by whole mount *in situ* hybridization that was performed as described [35] on 9.0 dpc mouse embryos using expressed sequence tag (ESTs) from Image clone 5716887 as a probe for *Cyr61*.

### Accession Numbers

The NCBI EntrezGene (http://www.ncbi.nlm.nih.gov/sites/entrez/query.fcgidbgene) accession numbers for the genes discussed in this paper are *Axin2* (12006), *c-Myc* (17869), *Cyr61* (16007), *Dact1* (59036), *Dkk1* (13380), *Fgf9* (14180), *Gbx2* (14472), *Hoxb1* (15407), *Lfng* (16848), *Rho* (212541) and *Spen* (56381).

## Supporting Information

Figure S1 (3.25 MB PDF)

Table S1 (0.02 MB XLS)

Table S2 (0.06 MB XLS)

Table S3 (0.07 MB XLS)

Table S4 (0.06 MB XLS)

Table S5 (0.06 MB XLS)

Table S6 (0.06 MB XLS)

Table S7 (0.02 MB XLS)

Table S8 (0.25 MB XLS)

Table S9 (0.13 MB XLS)

Table S10 (0.13 MB XLS)

Table S11 (0.13 MB XLS)

Table S12 (0.13 MB XLS)
