# Supplementary material for: Comparison of Pattern Detection Methods in Microarray Time Series of the Segmentation Clock
Source: PLoS One. 2008 Aug 6;3(8):e2856. doi: 10.1371/journal.pone.0002856 (PMC2481401; doi:10.1371/journal.pone.0002856)
Supplement: Figure S1 — (3.25 MB PDF) [file pone.0002856.s001.pdf]

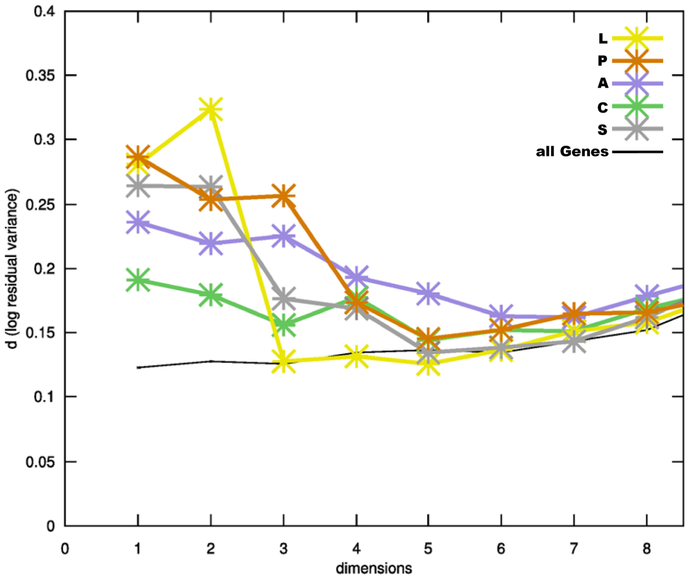

Figure S1. Dimensionality analysis of the top 300 datasets inferred from principal component analysis. The plot shows changes in residual variance as subsequent principal components are taken into account. Intrinsic dimensions in each set are considered significant until differences in log residual variance drop and converge towards the common noise level. L has two significant degrees of freedom, corresponding to sine and cosine of the main harmonic oscillation. S has two strong sin-cos components, plus two additional, presumably related to shape parameters. P has three degrees of freedom (corresponding to difference between phase 1 and 3, difference between phase 2 and 3, and variation within phase 3). The high number of degrees of freedom (between 4 and 6) in C and A stem from the uninformed priors used in these methods allowing for discovery of unrestricted profile shapes. **L**, Lomb-Scargle analysis; **P**, Phase consistency; **A**, Address reduction; **C**, Cyclohedron test; **S**, Stable persistence.
